# The mediating role of resilience between perceived social support and sense of security in medical staff following the COVID-19 pandemic: A cross-sectional study

**DOI:** 10.3389/fpsyt.2023.1096082

**Published:** 2023-03-07

**Authors:** Qingxia He, Peng Xu, Huajun Wang, Shibin Wang, Lulu Yang, Zhiqiong Ba, Huigen Huang

**Affiliations:** ^1^Guangdong Mental Health Center, Guangdong Provincial People's Hospital (Guangdong Academy of Medical Sciences), Southern Medical University, Guangzhou, China; ^2^Nursing Department, Guangdong Provincial People's Hospital (Guangdong Academy of Medical Sciences), Southern Medical University, Guangzhou, China

**Keywords:** resilience, sense of security, perceived social support, mediating effect, medical staff, structural equation model, COVID-19

## Abstract

**Background:**

The COVID-19 pandemic not only posed a serious threat to public life and health but also had a serious impact on people's mental health, especially that of medical staff. Perceived social support is an important factor in one's sense of security.

**Objective:**

Following the COVID-19 pandemic, the goal is to explore the potential mediating role of resilience in the relationship between perceived social support and the sense of security of Chinese medical personnel.

**Methods:**

The multi-stage proportionally stratified convenience sampling method was adopted to select 4,076 medical professionals from 29 hospitals in Guangdong Province between September 2020 and October 2020. The Sense of Security Scale for Medical Staff, the Chinese version of the Connor-Davidson Resilience Scale, and the Perceived Social Support Scale were employed in this study. For statistical analysis and structural equation modeling (SEM), the SPSS 23.0 and Amos 24.0 software packages were used. Regression analysis was used to select the control variables to be included in the SEM. SEM analysis was conducted to verify the mediating effect of resilience on the relationship between perceived social support and a sense of security.

**Results:**

Pearson's correlation analysis showed that perceived social support and resilience were positively associated with a sense of security (correlation coefficients range from 0.350 to 0.607, *P* < 0.01), and perceived social support (correlation coefficients range from 0.398 to 0.589, *P* < 0.01) was positively associated with resilience. Structural equation modeling revealed that resilience played a partial mediating role in the association between perceived social support and a sense of security (60.3% of the effect of perceived social support on security was direct, and 39.7% of the effect was mediated by resilience).

**Conclusions:**

Hospital managers should make efforts to develop resilience. Interventions based on resilience should be developed to enhance the perception of social support and strengthen one's sense of security.

## 1. Introduction

On 30/01/2020, the World Health Organization (WHO) declared the coronavirus outbreak an international health emergency (1). According to the United Nations, the COVID-19 pandemic caused not only physical but also psychological harm (2). With the increasing workload of pandemic wards, the psychological stress of medical staff gradually changed from acute to chronic, and due to forced quarantine and nationwide lockdowns, it manifested in various ways (3). As front-line medical personnel, physicians and nurses experienced the fear associated with a novel infectious disease and close contact with people who had tested positive, and they had to work under extreme pressure to diagnose, treat, and care for COVID-19 patients (4). Such circumstances would put them at high risk of developing mental health problems. It is particularly important to understand the mental health status of medical personnel during this period.

A sense of security is defined as a feeling of confidence, safety, and freedom from fear and anxiety, especially the feeling of being able to meet one's various needs now and in the future (5). Security can be seen as a proxy for mental health (6). Recently, security in the workplace has grown in popularity, particularly in medical settings. Medical personnel spend the majority of their time at medical facilities. These professionals' sense of security can reflect their level of affirmation and corresponding attitude toward patients, their families, and the medical setting (7). At present, medical staff security is highly valued, and the Chinese government has enacted the first law to protect their safety.

Medical staff serve as health guardians and play a vital role in global health systems. Chinese healthcare workers are increasingly important to policymakers hoping to achieve the Healthy China 2030 targets. However, healthcare workers face low social support at work, overtime, high workloads, and exposure to harmful body fluids, odors, and noises, which can lead to job insecurity (8, 9). Insecurity is a real problem for healthcare workers and the world's healthcare systems (10). A lack of security has been associated with adverse effects on a wide range of physical and mental health outcomes. For example, exposure to job insecurity not only leads to job burnout and resignation behaviors, but it can also damage the quality of life of medical staff (11, 12). There are significant negative correlations between depressive symptoms and job security (13). Furthermore, a lack of security is associated with physical discomfort (14). The issues described made daily work and therapeutic relationships difficult, and professionals were unable to provide quality care (15). For these reasons, it is very necessary to prioritize the sense of security for medical staff.

Experiencing social support is a subjective feeling that represents the sum of all social support perceived by the individual (16). According to the main effect model, social support influences mental health positively (17). Indeed, according to some researchers, the higher the level of perceived social support, the better the mental health status (18). Moreover, perceived social support is an important strategy to reduce anxiety (19). Additionally, it is thought that perceived social support is a predictor of depression (20). As a result, the perception of social support is critical to the mental health of medical staff.

The term “resilience” refers to the ability and quality of an individual to adapt to a changing environment (21). Some studies suggest that resilience acts as a buffer against stress (22). In the case of medical personnel, resilience is expressed in their ability to demonstrate perseverance and good self-control by constantly adapting and adjusting to difficulties and pressure (23). The greater the level of mental resilience, the more confidence and courage an individual has in dealing with setbacks (24). The higher the level of mental health and the greater the psychological resilience of medical personnel, the less vulnerable they are to other events (25, 26).

According to the job demand-resource model, all job characteristics can be divided into two categories: resources and demands. Social support can be regarded as a job resource and an important predictor of security (27). Psychological resilience is an individual resource. When individuals feel supported by their organization and by their friends and family, they have a greater sense of security, which further increases when an individual maintains a high level of psychological resilience at the same time. Previous research has shown that perceived social support is positively associated with a sense of security (28). Studies have reported that resilience is related to a sense of security (29). Meanwhile, social support is an important psychological resilience protective factor (30). To summarize, there is a close connection between perceived social support, a sense of security, and resilience, indicating that resilience influences perceived social support and security. Therefore, this study will consider resilience as a possible mediator in the relationship between perceived social support and a sense of security. Furthermore, developing strategies to increase the perception of social support can help reduce negative mental health outcomes by elevating the sense of security.

## 2. Material and methods

### 2.1. Study design and participants

A cross-sectional design was used in this study. We collected data between September 2020 and October 2020. The samples were obtained using a multi-stage proportionally stratified convenience sampling procedure. In the first stage, hospitals were sampled according to the sampling ratio ≈2:1:1 (tertiary hospital: secondary hospital: primary hospital). The sampling ratio was determined according to the number of tertiary, secondary, and primary public hospitals in Guangdong Province and the composition ratio of hospitals in the four regions of Guangdong Province. In the second stage, medical staff (doctors, nurses, pharmacists, and technicians) were sampled according to the sampling ratio ≈4:8:1:1 based on the number of doctors, nurses, pharmacists, and technicians in Guangdong Province. Participants were eligible if they were (a) registered or licensed medical staff or (b) had more than 1 year of experience working in healthcare. Medical personnel who were off duty, suffering from mental illness, or who were unwilling to participate in the study were excluded. To achieve 80% power, with alpha set at 0.05, a sample size of 247 medical staff would be required (31, 32). Considering a possible non-response rate of 20%, the final sample was required to be at least 309. A total of 4,173 questionnaires were distributed, with 4,076 (97.68%) validly returned.

### 2.2. Data collection

The survey was completed through an online survey platform (“SurveyStar,” Changsha Ranxing Science and Technology, Shanghai, China) that did not allow respondents to take the survey more than once. We contacted hospital managers *via* email with an invitation to participate in this study. Once they agreed, they were asked to send the work group. The researchers explained the goals of the study to the participants and assured them that the data would be kept confidential. After they decided to participate in the study, informed consent was obtained. Only those who agreed to participate and completed the questionnaire could submit it.

### 2.3. Measurements

#### 2.3.1. Perceived social support scale

The 12-item PSSS was developed by Zimet (33) and translated into Chinese by Jiang, and it included three dimensions: Family support (four items), Friend support (four items), and Other support (four items). Items were rated on a seven-point scale ranging from 1 (strongly disagree) to 7 (strongly agree). The greater the mean score of these 12 items, the greater one's perception of social support. The total Cronbach's α coefficient on the scale was 0.94. In this study, Cronbach's α coefficient scale was 0.934.

#### 2.3.2. A Chinese version of the Connor-Davidson Resilience Scale

The Connor-Davidson Resilience Scale (CD-RISC) was developed by Connor and Davidson (34) and Yu and Zhang confirmed the validity and reliability of its Chinese version (35). The scale comprised 25 items, each of which was rated on a five-point Likert scale, with scores 0–4 being judged as “not true at all” to “nearly always true.” The CD-RISC also included three subscales: Tenacity, Strength, and Optimism. Total points range from 0 to 100, with a higher score indicating greater resilience. The Chinese version's Cronbach's α coefficient was 0.91 for the total score, and 0.88, 0.80, and 0.60 for the three subscales. Cronbach's α coefficient in the current study was 0.912.

#### 2.3.3. Sense of security scale for medical staff

The scale was developed by the research group in the early stages, with a total of 22 items, including five dimensions: Environment, Patients, Self, Organizational management, and Social support (36). The Likert 5 rating method is used, with all items being reverse scored on a 5-point scale ranging from 1 (strongly agree) to 5 (strongly disagree). A higher score means a greater level of security. The total Cronbach's α coefficient on the scale is 0.939. Cronbach's α coefficients in all dimensions are all >0.7, test-retest reliability is 0.808, and the split-half reliability coefficient is 0.967. The scale shows good psychometric properties. In this study, Cronbach's α coefficient is 0.956. The scale's total score is obtained by adding the scores of all the items. The minimum total scale score is 22 and the maximum is 110.

### 2.4. Participants' demographic characteristics

Gender, age, work experience (in years), educational level (junior college or below; bachelor's degree; doctorate or higher), job position, marital status (married; single; divorced or separated), and hospital level were all collected demographic characteristics. The study population was divided into four age groups: ≤ 30, 31–35, 36–40, and ≥41 years. The participants were divided into four work experience groups: 1–5, 6–10, 11–20, and ≥21 years. The positions include doctors (whose job is to treat people who are ill or injured), nurses (whose job is to provide care for people who are ill or injured), pharmacists (whose job is to prepare and distribute hospital medications), and technicians (whose job is to support the hospital's clinical departments). Three types of hospitals were considered: tertiary hospitals, secondary hospitals, and primary hospitals. Tertiary hospitals are the largest type, typically with more than 500 beds and cutting-edge technology. Primary hospitals are the smallest hospitals, usually with fewer than 100 beds and limited technology. Apart from tertiary hospitals and primary hospitals, the others belong to the Secondary hospitals.

### 2.5. Statistical analyses

For statistical analysis, SPSS Statistics version 23.0 (SPSS Inc., Chicago, IL, USA) and AMOS version 24.0 (IBM, Armonk, NY, USA) were used. SPSS was used for descriptive statistics, Pearson's correlation, and regression analysis. AMOS was used for structural equation modeling (SEM). SEM is a method for specifying and testing linear relationship models between observed and latent variables. Regression analysis was used among the demographic variables to select the control variables to be included in the SEM.

In order to obtain the model, the first phase included confirmatory factor analysis (CFA) of the Perceived Social Support Scale (PSSS), Connor-Davidson Resilience Scale (CD-RISC), and Sense of Security Scale for Medical Staff (SSS-MS). The internal reliability of the measurement tools was tested using Cronbach's α coefficient and composite construct reliability, and its validity was examined by CFA.

The SEM employed the maximum likelihood estimation method, and the model's goodness-of-fit indices were evaluated through relative and absolute indices, which included the chi-square value, chi-square degree of freedom (χ^2^/df), goodness-of-fit index (GFI), adjusted goodness-of-fit index (AGFI), root mean square error of approximation (RMSEA), comparative fitting index (CFI), normed fit index (NFI), Tucker-Lewis index (TLI), and the incremental fit index (IFI). The following values were regarded as acceptable: χ^2^/df < 3, GFI > 0.90, AGFI > 0.90, CFI > 0.90, NFI > 0.90, TLI > 0.90, RMSEA ≤ 0.08 (37) and IFI > 0.90 (38).

### 2.6. Ethical considerations

This study was approved by the Guangdong Provincial People's Hospital Ethics Committee (KY2020-579-01). Before implementation, the respondents were informed of the research objectives and signed an informed consent form, which clearly stated that the survey would be completed anonymously. The data obtained were protected by the researchers in order to maintain confidentiality and prevent any potential unintended uses.

## 3. Results

### 3.1. Descriptive statistics

The sample consisted of 4,076 licensed and registered medical professionals (doctors, nurses, pharmacists, and technicians) from 29 public hospitals, which included 14 tertiary hospitals, eight secondary hospitals, and seven primary hospitals spread across the four regions (Pearl River Delta, eastern Guangdong, western Guangdong, and northern Guangdong) of Guangdong Province. Of the 4,076 medical staff, 1,019 (25.0%) were doctors, 2,238 (54.9%) were nurses, 438 (10.7%) were pharmacists, and 381 (9.3%) were technicians. Among them, 231 (5.7%) were from primary hospitals, 1,462 (35.9%) were from secondary hospitals, and 2,383 (58.5%) were from tertiary hospitals. The average age of the 4,076 participants was (32.46 ± 9.06) years, with 1,484 (36.4%) men and 2,592 (63.6%) women. In terms of education, 1,781 (43.7%) had a junior college or lower degree, 1,363 (33.4%) had a bachelor's degree, and 943 (22.9%) had a master's degree or higher. The other demographic characteristics of this sample have been presented in Table 1.

**Table 1:** Sociodemographic characteristics of medical staff (*N* = 4,076).

**Variables**	**Categories**	***N***	**%**
Gender	Men	1,484	36.4
	Women	2,592	63.6
Age (in years)	≤ 30	2,129	52.2
	31–35	686	16.8
	36–40	494	12.1
	≥41	767	18.8
Work experience (in years)	1–5	1,376	33.8
	6–10	1,172	28.8
	11–20	882	21.6
	≥21	646	15.8
Educational level	Junior college or lower	1,781	43.7
	Bachelor's degree	1,363	33.4
	Master's degree or higher	943	22.9
Job position	Doctor	1,019	25.0
	Nurse	2,238	54.9
	Pharmacist	438	10.7
	Technician	381	9.3
Marital status	Married	2,655	65.1
	Single	1,339	32.9
	Divorced/separated	82	2.0
Hospital level	Primary hospital	231	5.7
	Secondary hospital	1,462	35.9
	Tertiary hospital	2383	58.5

### 3.2. Model identification

Standardized factor loading in measurement mode should be between 0 and 1, with higher values suggesting better indications of the observed variables for the latent variables. To represent the validity and reliability of each construct, three measurements specific to SEM were used in this study: factor loading, composite reliability (CR), and average variance extract (AVE). The CRs of all the constructs were >0.85 (as shown in Table 2), signifying that the items were satisfactory indicators. Furthermore, based on the CFA results, the AVE values showed that all of the latent variables had sufficient convergent and discriminant validity.

**Table 2:** Reliability and validity test of the measurement model (*N* = 4,076).

**Scale**	**Dimension**	**M ±SD**	**Factor loading**	**Cronbach's alpha**	**AVE**	**CR**
Perceived social support scale	Family support	17.67 ± 6.18	0.868	0.872	0.708	0.879
	Friend support	17.61 ± 5.88	0.876	0.865		
	Other support	15.93 ± 5.99	0.777	0.883		
Connor-Davidson resilience scale	Tenacity	27.38 ± 8.06	0.879	0.867	0.676	0.861
	Strength	17.55 ± 4.61	0.862	0.759		
	Optimism	8.29 ± 2.84	0.716	0.622		
Sense of security scale for medical staff	Environment	10.83 ± 4.02	0.788	0.827	0.644	0.900
	Patients	11.80 ± 4.45	0.663	0.921		
	Self	9.30 ± 3.13	0.790	0.799		
	Organizational management	21.37 ± 7.41	0.885	0.920		
	Social support	11.55 ± 4.60	0.868	0.902		

CR, composite reliability; AVE, average variance extracted.

### 3.3. Relationships between medical staff members' perceptions of social support, resilience, and sense of security

The correlation analysis results showed that each PSSS subscale was positively and significantly related to resilience and five dimensions of the SSS-MS (r ranged from 0.381 to 0.589, *P* < 0.01). Of the three dimensions of perceived social support, the correlation between other support (such as from coworkers and supervisors) and security is the strongest, followed by family support and friend support. Resilience had a positive and significant association with each subscale and total score of the SSS-MS, respectively (r ranged from 0.350 to 0.607, *P* < 0.01; Table 3).

**Table 3:** Correlations among observed indicators in the SEM (*N* = 4,076).

	**Sense of security scale for medical staff**						**Connor-Davidson resilience scale**				**Perceived social support scale**			
	**1**	**2**	**3**	**4**	**5**	**6**	**7**	**8**	**9**	**10**	**11**	**12**	**13**	**14**
1	–													
2	0.623^**^	–												
3	0.638^**^	0.545^**^	–											
4	0.674^**^	0.525^**^	0.713^**^	–										
5	0.673^**^	0.558^**^	0.657^**^	0.794^**^	–									
6	0.840^**^	0.752^**^	0.817^**^	0.912^**^	0.882^**^	–								
7	0.467^**^	0.428^**^	0.470^**^	0.539^**^	0.531^**^	0.582^**^	–							
8	0.418^**^	0.393^**^	0.428^**^	0.498^**^	0.488^**^	0.532^**^	0.759^**^	–						
9	0.372^**^	0.350^**^	0.368^**^	0.443^**^	0.442^**^	0.474^**^	0.618^**^	0.627^**^	–					
10	0.483^**^	0.447^**^	0.487^**^	0.565^**^	0.557^**^	0.607^**^	0.953^**^	0.895^**^	0.766^**^	–				
11	0.421^**^	0.398^**^	0.436^**^	0.478^**^	0.461^**^	0.522^**^	0.416^**^	0.418^**^	0.335^**^	0.446^**^	–			
12	0.414^**^	0.381^**^	0.412^**^	0.460^**^	0.454^**^	0.505^**^	0.415^**^	0.419^**^	0.341^**^	0.447^**^	0.769^**^	–		
13	0.449^**^	0.437^**^	0.465^**^	0.505^**^	0.481^**^	0.555^**^	0.366^**^	0.370^**^	0.306^**^	0.395^**^	0.661^**^	0.678^**^	–	
14	0.478^**^	0.453^**^	0.489^**^	0.537^**^	0.519^**^	0.589^**^	0.445^**^	0.449^**^	0.366^**^	0.479^**^	0.907^**^	0.909^**^	0.870^**^	–

1, Environment; 2, Patients; 3, Self; 4, Organizational management; 5, Social support; 6, Sense of security scale for medical staff; 7, Tenacity; 8, Strength; 9, Optimism; 10, Connor-Davidson resilience scale; 11, Family support; 12, Friend support; 13, Other support; 14, Perceived social support scale. ^**^P < 0.01 (two-sided).

### 3.4. Mediation analysis of perceived social support, psychological resilience, and sense of security

**Figure 1:**
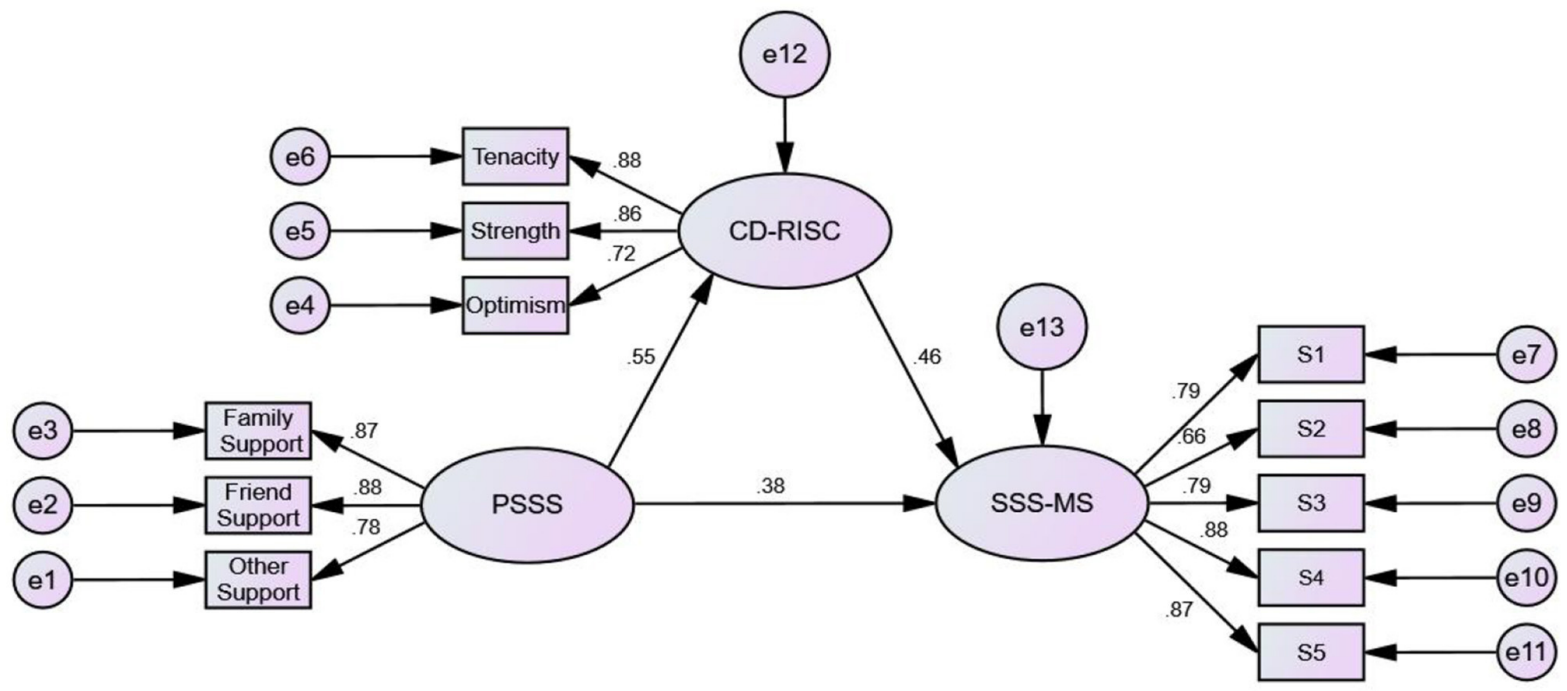
Structural equation model of perceived social support, resilience, and sense of security (Standardized). S1, Environment; S2, Patients; S3, Self; S4, Organizational management; S5, Social support.

The model explained 55.6% of the variance in the general sense of security. Figure 1 shows the final model with standardized path coefficients, and the unstandardized and standardized path coefficients for the model are presented in Table 4. Regression analysis was used to test the effects of demographic characteristics on the sense of security. In the demographic characteristics, only educational level (β = 0.037, *P* < 0.001), job position (β = 0.037, *P* < 0.001), and marital status (β = −0.115, *P* < 0.001) had a significant effect on the sense of security, which was addressed as a control variable later in the final model. The model fit showed that χ^2^/df = 1.02 (< 3), RMSEA = 0.01 (< 0.08), and the relative and absolute indices values were all >0.90.

**Table 4:** Unstandardized and standardized path coefficients for the structural model (*N* = 4,076).

	**B**	**β**	**S.E**.	**C.R**.	***P***
PSSS → CD-RISC	0.239	0.547	0.008	28.776	^***^
CD-RISC → MS-SSS	0.709	0.457	0.028	24.960	^***^
PSSS → MS-SSS	0.257	0.378	0.012	21.926	^***^
Educational level → MS-SSS	0.149	0.037	0.048	3.122	0.002
Job position → MS-SSS	0.053	0.037	0.017	3.130	0.002
Marital status → MS-SSS	−0.248	−0.115	0.026	−9.585	^***^

PSSS, Perceived social support scale; CD-RISC, Connor-Davidson resilience scale; MS-SSS, Sense of security scale for medical staff. B, unstandardized path; β, standardized path; S.E., standard error; C.R., critical ratio; P, significance level. ^***^P < 0.001.

As seen in Figure 1, perceived social support was positively related to a sense of security (β = 0.378, *P* < 0.001), perceived social support was positively related to resilience (β = 0.547, *P* < 0.001), and resilience was positively related to a sense of security (β = 0.457, *P* < 0.001). Additionally, perceived social support had an indirect protective effect on the sense of security as it influenced resilience (β = 0.547^*^0.457 = 0.249, *P* < 0.001), which in turn led to a significant effect on the sense of security. As a result, the total effect of perceived social support against security was 0.627. The results indicated that resilience played a partial mediating role in the relationship between perceived social support and a sense of security. The ratio of mediating effect to total effect was 39.7%. In other words, psychological resilience mediated 39.7% of the effect of perceived social support on security, while 60.3% was a direct effect.

## 4. Discussion

The main purpose of this study was to explore the potential mediating role of resilience in the association between perceived social support and a sense of security among Chinese medical staff following the COVID-19 pandemic. Furthermore, perceived social support was a significant predictor of a sense of security. Our study found that perceived social support, sense of security, and resilience are all significantly positively correlated with one another. According to the results, perceived social support not only directly affects medical personnel's sense of security but also indirectly influences it *via* a partial mediating role of resilience. The mediating effect accounts for 39.7% of the total effect.

Previous research identified a variety of security-related factors, such as interpersonal relationships [e.g., individual characteristics (39), coping style (40), and perceived social support (41, 42)]. While perceived social support is the most commonly reported predictor, it was found that perceived social support can significantly predict a sense of security and that having a higher level of perceived social support is an important factor in maintaining and improving individual security (42). In addition, studies have found that perceived social support is an effective way to cope with job insecurity and can decrease its negative effects (43) because social support is a powerful “shield” against the experience and consequences of work-related stress (44). On the one hand, the higher the level of perceived social support for an individual, the easier it is to get support and help from the outside when encountering difficulties (45). During the lockdown, most people worked online, and all families lived together as family units, sharing prevention and control information. Most likely because healthcare workers can feel the spiritual and material support provided by family, friends, and others (colleagues, leadership, relatives, etc.) during the COVID-19 pandemic. These social support can meet the need for their self-esteem, love, and interpersonal communication (46), so as to reduce the negative impact of stressful experiences on medical staff (47), which is conducive to their physical and mental comfort and maintaining their sense of security. On the contrary, with insufficient social support, medical staff will feel helpless in the face of stress and difficulties, and the need for self-esteem, love, and a sense of belonging will not be met, resulting in a decrease in security (48). Furthermore, our findings indicate that other supports had the strongest correlation with the sense of security of medical professionals. This was consistent with previous studies. For instance, Yang (49) and Ajnakina (14) discovered that low social support from coworkers and supervisors was associated with the greatest decrease in the sense of security. Such support can serve as a valuable work resource and help medical staff maintain their sense of security (50). Higher levels of social support from colleagues and supervisors act as a protective factor against workplace violence (51). Research has found that healthcare workers need support from their organizations and colleagues to reduce stress, and poor relationships at work can lead to poor judgment (52). Employees not only value instrumental support in material and other aspects but also need intimate and respectful social-emotional support (53), which can help meet their social and psychological needs. When medical staff is given adequate leadership support, such as increased pay levels, increased promotion opportunities, and learning opportunities, they tend to feel secure enough (54). Employees will have positive emotions when they receive sufficient emotional support, such as communication between superiors and subordinates, positive feedback from superiors, and emotional incentives, thus increasing their positive evaluation of work feelings (55).

We found that improved perceived social support provides the medical staff with more psychological resilience, which further promotes their sense of security. Until now, few studies have analyzed the impact of resilience on perceived social support and a sense of security among healthcare workers. Security was once considered as important as mental health. There was some evidence that the relationship between perceived social support and mental health is mediated by resilience in nursing students (56) and junior middle school students (57). On the one hand, resilience was found to be a positive contributor to the sense of security in Chinese medical staff (29), suggesting that higher levels of resilience result in a greater sense of security (22). This indicates that medical professionals with higher mental resilience have more positive energy and can deal with stressful events more actively, so their level of security is also higher (58). Healthcare workers are particularly vulnerable to a lack of security because of the complexity of their work and the fear of being infected during the COVID-19 pandemic. When medical staff have a high level of mental resilience, they can maintain a positive attitude toward themselves even when faced with negative events such as the pressure of professional promotions and the conflicts between doctors and patients, allowing them to retain a high level of security (59). This difference may be due to the fact that, in the fight against COVID-19, healthcare workers are still improving their sense of security and resilience despite a new and complex working environment, which includes adjusting to heavy protective equipment and a high-intensity workload. Furthermore, perceived social support has been shown to protect and promote mental resilience (30). This finding was consistent with a previous study, which showed that perceived social support can protect and promote mental resilience (60). Medical personnel can get social support from family, friends, supervisors, and colleagues. Family and friends can understand and emotionally support these healthcare workers. In addition to the care of supervisors and colleagues, their encouragement can effectively alleviate their adverse mood (61). This can be used as a stress buffer factor (62), effectively lessening the emotional stimulation brought on by medical work so as to maintain these professionals' mental resilience. When medical workers have more social support, they will have more confidence and courage to deal with difficulties and setbacks at work, and they will be able to better adjust to the impact of adverse factors, promoting the development of their mental resilience (22), and thus improving their job security in the face of pressure. This supports our findings that perceived social support works not only on its own but also in combination with resilience.

The exploration of the relationship among perceived social support, resilience and sense of security has provided new insights for hospital administrators in managing perceived social support and a sense of security among medical staff. The findings revealed that perceived social support can help medical professionals identify the meaning of their work, increase their resilience, and strengthen their impact on the sense of security. The findings suggest that hospital managers may facilitate the resilience of medical staff, which may be an effective method to improve their sense of security. Meanwhile, reasonable evidence for the resilience-enhancing effects of workshops and cognitive behavioral interventions has been discovered (63). Some interventions, such as psychological skills training interventions, communication skills training, and mindfulness-based interventions, have been shown to improve the resilience of healthcare professionals (64, 65).

## 5. Conclusions

The COVID-19 pandemic continues to spread around the world. However, it is accompanied by an increase in people's sense of insecurity. The problems resulting from a low sense of security may pose a major psychological threat to medical personnel. It is necessary to be aware of the adverse consequences caused by a reduced sense of security. This study indicated that perceived social support was significantly associated with healthcare workers' sense of security, and resilience mediated the relationship between this and perceived social support. Our findings suggest that perceived social support may play an important role in increasing one's sense of security. Hospital managers should make efforts to develop perceived social support. Interventions based on perceived social support should be developed to increase resilience and strengthen one's sense of security.

## 6. Limitations

This study has some limitations. Firstly, this is a cross-sectional study that only analyzed the relationship among perceived social support, resilience, and sense of security. However, the causality between the core variables in our study could not be determined. A future longitudinal study will be required to confirm this. Secondly, convenience sampling limits the generalizability of the results. However, the 29 hospitals we selected are distributed in the four regions of Guangdong Province: Pearl River Delta, eastern Guangdong, western Guangdong, and northern Guangdong, and the sampling was carried out according to the proportion of the number of hospitals covered by the four regions. At the same time, the sampling ratio of doctors, nurses, pharmacists, and technicians is strictly adhered to. Therefore, the representativeness of the sample can be ensured by considering both proportional samplings of hospitals and medical staff. Finally, due to time and financial restraints, all measurements are self-reported. It is still possible for information bias to have occurred due to over- and under-reporting. Although all the scales are self-reported, they all have good psychometric properties and are widely recognized and used. Despite these limitations, the current study contributes to the previous literature both theoretically and practically. Theoretically, this study adds to the previous research by exploring the mediation model, which would help further understand the relationship between perceived social support and a sense of security. Practically, these findings are essential for improving the sense of security among healthcare workers.

## Data availability statement

The original contributions presented in the study are included in the article/supplementary material, further inquiries can be directed to the corresponding author.

## Ethics statement

The studies involving human participants were reviewed and approved by Guangdong Provincial People's Hospital Ethics Committee. The patients/participants provided their written informed consent to participate in this study.

## Author contributions

QH: conception of the study, data analysis, and drafting of the manuscript. HH: study design, critical review, and revision of the manuscript. PX, HW, and SW: data collection, review, and revision of the manuscript. LY and ZB: data collection and review of the manuscript. All authors contributed to this study and approved the final version of the manuscript.
